# Surface Acoustic Wave (SAW) Sensors for Hip Implant: A Numerical and Computational Feasibility Investigation Using Finite Element Methods

**DOI:** 10.3390/bios13010079

**Published:** 2023-01-02

**Authors:** Muhammad Hafizh, Md Mohiuddin Soliman, Yazan Qiblawey, Muhammad E. H. Chowdhury, Mohammad Tariqul Islam, Farayi Musharavati, Sakib Mahmud, Amith Khandakar, Mohammad Nabil, Erfan Zal Nezhad

**Affiliations:** 1 Department of Mechanical and Industrial Engineering, Qatar University, Doha 2713, Qatar; 2 Department of Electrical, Electronic and Systems Engineering, Faculty of Engineering and Built Environment, Universiti Kebangsaan Malaysia, Bangi 43600, Selangor, Malaysia; 3 Department of Electrical Engineering, Qatar University, Doha 2713, Qatar; 4 Centre for Advanced Electronic and Communication Engineering, Department of Electrical, Electronic and Systems Engineering, Faculty of Engineering & Built Environment, Universiti Kebangsaan Malaysia (UKM), Bangi 43600, Selangor, Malaysia; 5 Department of Biomedical Engineering, University of Texas at San Antonio, San Antonio, TX 78249, USA

**Keywords:** hip implant, surface acoustic wave (SAW), numerical analysis, finite element method, total hip implant replacement

## Abstract

In this paper, a surface acoustic wave (SAW) sensor for hip implant geometry was proposed for the application of total hip replacement. A two-port SAW device was numerically investigated for implementation with an operating frequency of 872 MHz that can be used in more common radio frequency interrogator units. A finite element analysis of the device was developed for a lithium niobate (LiNBO3) substrate with a Rayleigh velocity of 3488 m/s on COMSOL Multiphysics. The Multiphysics loading and frequency results highlighted a good uniformity with numerical results. Afterwards, a hip implant geometry was developed. The SAW sensor was mounted at two locations on the implant corresponding to two regions along the shaft of the femur bone. Three discrete conditions were studied for the feasibility of the implant with upper- and lower-body loading. The loading simulations highlighted that the stresses experienced do not exceed the yield strengths. The voltage output results indicated that the SAW sensor can be implanted in the hip implant for hip implant-loosening detection applications.

## 1. Introduction

The hip is an essential anatomical structure that connects the upper and lower body for people to perform activities while standing up. The hip joint supports the weight of the body and provides the stability required in the human structure. Nevertheless, deterioration of the joint through wear and age often requires hip joint arthroplasty or total hip replacements (THR). The National Joint Registry (NJR) documented 138,0472 hip replacements, of which 90% were initial replacements, and 10% were revisions [1]. In addition, 42.3% and 14.7% of implant revisions were performed due to implant aseptic loosening and dislocation. Nevertheless, THR is not guaranteed to function well immediately, and its limited life span means that patient monitoring post-operation is important for the longevity and success of the implant. A subpar total hip replacement can lead to longer recovery time and risky corrective procedures. To avoid unwanted clinical complications, early diagnosis of implant loosening is required [2]. Presently, medical trials and radiography are employed for loosening diagnostics. The accuracy of radiographs is not very good, despite the advanced state of radiography technology. Approximately 80% sensitivity and specificity have been found for radiography [3]. In certain situations, it is challenging to get good pictures of the loosening locations using bone scintigraphy, according to studies found in [4]. Implantable sensors for biomedical applications can provide an accurate and long-term solution to measurement and monitoring in comparison to multiple conventional invasive measurement procedures. Complications from total hip replacements can be monitored to reduce the impact on the patient, since wear, dislocation and impingement values are not being actively monitored [5].

Surface acoustic wave (SAW) devices are piezoelectric-based devices that use interdigital transducers (IDT) to generate waves that propagate over a piezoelectric substrate [6,7]. SAW devices have already been used in many commercial applications, taking advantage of compact size, low manufacturing cost, robust operation in harsh environments and ability to operate passively and wirelessly by connecting an antenna to the IDT input where a radio frequency (RF) pulse is detected [8,9]. Thus, no power supply or battery is required to drive the sensor, which is extremely agreeable for implant applications [9]. With SAW sensors, loosening and dislocation problems of surgeries can optimise the function of the joint biomechanically and ensure long-term success. Additionally, remote-sensing technology has continued to mature in recent decades with solutions to overcome power constraints. Nowadays, the development of bioinert and biocompatible materials that can be fabricated for artificial implants and bone replacements is becoming increasingly important [10,11,12]. 

**Figure 1:**
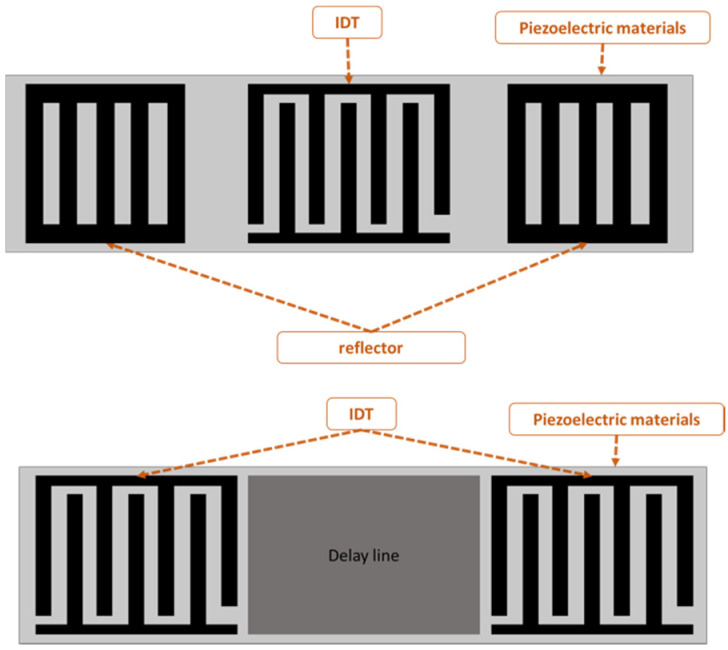
One- and two-port surface acoustic wave (SAW) devices.

SAW-devised technology offers promising performance in many applications, including pressure measurement, temperature measurement, strain measurement and filtering signals as a sensor. The SAW sensor converts physical quantitates directly to a change in time and frequency domain. The SAW sensor is classified into two types: one-port and two-port architecture, as shown in Figure 1. A sensor with one port operates with an IDT located between two reflectors, and the reflectors are added to avoid interference patterns or reduce insertion losses. The two-port SAW device is built with two IDTs with a separation between them called the delay line, as shown in Figure 1. The IDT converts the received electrical signal into a mechanical signal that propagates over the piezoelectric material. Due to physical changes such as temperature, force and humidity, the mechanical signal changes. Therefore, the response will change. Finally, the IDT converts the rest of the mechanical signal back to an electrical signal. 

One-port configuration was utilised for chemical sensing and signal filtering [13]. The delay line area is commonly used as a sensing area to measure physical quantities, such as temperature, humidity and strain [14,15]. SAW sensors with antennas have been shown to effectively transduce pressure information across in vivo studies [10]. In addition to the mentioned applications, SAW sensors have also been used for health monitoring and process monitoring [16]. In a variety of applications, including implants and other biomedical applications, there is a need for a sensor that can operate passively and wirelessly [17,18]. Zou et al. [19] described a method for wirelessly querying surface acoustic wave (SAW) sensors implanted in the major pulmonary artery, where surface acoustic wave systems are used to monitor pressure. Bao et al. [20] designed a strain sensor using a structure that consists of AlN/Pt/Ti/SiO2 deposited on a silicon substrate. Despite the different substrates used such as quartz, langasite (LGS, La3Ga5SiO14), lithium niobate (LiNbO3) and zinc oxide (ZnO), LiNbO3 substrate has been widely applied to increase the device efficiency because of the high electro-mechanical coupling coefficient. Ren et al. [21] designed and simulated a SAW sensor with a LiNbO3 diaphragm as the sensing element with an operating frequency of 50 MHz. Gopalsami et al. [22] developed an implantable SAW microsensor for seizure incipient surveillance based on local temperature variations in the brain’s epileptogenic regions that develop before and during an epileptic event. Konno et al. [23] proposed a two-port SAW strain sensor operating at 384 MHz; the sensor was based on 128°YX LiNbO3. Fischerauer et al. [24] designed a strain sensor based on a two-port configuration designed for 99.8 MHz. 

Notably, the majority of hip implant loosening detection systems are based on temperature and force [25], acoustic emission [26,27] and vibration [28,29] and strain gauge sensors [30], whereby each detection system faces some major disadvantages that have inspired researchers to develop a hip implant-loosening detection system via a SAW sensor. Ramachandran et al. [31] utilised the acoustic emission technique as a tool to provide information on implant structural degradation. Burton et al. [30] presented a bio-compatible wireless inductive strain-sensing device to monitor bone-hosting implant development and strain sensitivity. Applying a vibration signal to the femoral head to assess the signal’s response is another hip-loosening detection method [28,32]. Accelerometer readings indicate implant loosening. In a secured prosthesis, just the driving frequency appears in the frequency domain analysis. Thus, bone and prosthesis accelerate as one unit. Additional components (harmonics) in the frequency spectrum suggest a loose prosthesis. In a variety of applications, including implants and other biomedical applications, there is a need for a sensor that can operate passively and wirelessly [33,34]. The implanted temperature sensor requires an external regulator to maintain a voltage below 5 V, as high-power consumption might affect temperature readings owing to the heat created by the telemetry circuit and eddy currents. Even though the hip implant-loosening detection system based on vibration and acoustic emission has several advantages, such as avoiding power coils or antenna within the patient’s body, it still has signal-damping and transmission issues, resulting in a faulty detection system.

Following the research gaps, there is still a research opportunity to build intrusive sensors with compact size, cheap manufacturing cost, robust operation in severe environments and the ability to work passively and wirelessly by attaching an antenna [35,36]. The SAW sensor system is a feasible alternative since it is a well-established sensor system that is frequently utilised in medical applications. SAW sensors overcome the limitations of previous hip implant-loosening detection sensors by providing long-term quartz durability and nontoxicity and the possibility to operate at substantially lower power levels, retaining accurate frequency estimation while functioning with extremely high Q sensors.

This paper is structured as follows: Section 2 outlines the analytical parameters for modelling the SAW Sensor. Section 3 focuses on the numerical and computational analysis of the sensor. Hip implants and integration of the sensor are shown in Section 4. Finally, the investigation is summarised in Section 5. Following the literature and research interest, the purpose of this study is to construct a SAW sensor system that can detect the loosening of a hip implant under dynamic loading circumstances.

## 2. Numerical Analysis of a SAW Sensor

**Figure 2:**
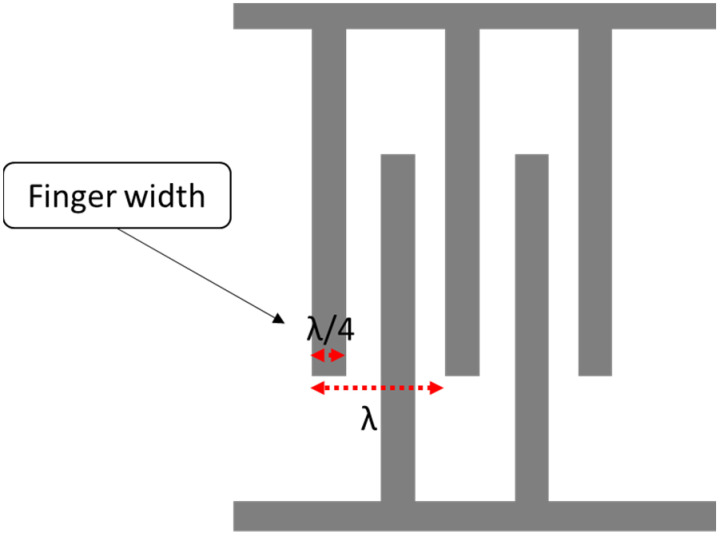
Interdigital transducer (IDT) layout.

The IDT is considered the main component of the SAW sensor. The IDT consists of two metal comb structure electrodes with the ability to operate in two modes: transmitter and receiver mode. The IDT operation principle is based on the electric field that is generated due to the applied voltage across IDT electrodes that are made of a combination of positive and negative electrodes with uniform lengths, widths and gaps. However, some applications have used non-uniform dimensions. Non-uniform size can generate higher harmonic waves. The area between fingers accumulates charge and produces an electric field, which leads to periodic expansion and compression and creates a strain. The summation of all strains generated from each period creates a larger wave called the surface acoustic wave (SAW). The wavelength is equal to the pitch of the electrodes. The operating frequency derived from the wave equation can be represented as:

(1) $$\lambda =\frac{v}{f_{0}}$$

where v is the acoustic velocity in the medium, and$\;f_{0}\;$is the operating frequency of the SAW device. The width of a single IDT finger is given by *λ*/4, so the IDT finger width affects the operating frequency. The smaller the width, the higher the operating frequency, as shown in Figure 2. In terms of the IDT material, substrate adhesion, the boiling point, resistivity and cost are properties to consider during the selection. A good surface substrate adhesion is critical for achieving a good coupling between the IDT and piezoelectric substance. The boiling point determines the process used for fabricating the SAW device.

Copper is a commonly used material for IDT for several applications. However, it tends to diffuse into the substrates common to microfabrication, and it has high resistivity. On the other hand, copper shows a lower insertion loss. Other materials for IDT have been tested and showed a lower resistivity, such as aluminium and chromium. Another important factor is the frequency shift from centre frequency during operation. Aluminium is more efficient for the excitation of the sensing structure and has good substrate adherence [37]. Piezoelectric materials are commonly used to fabricate SAW-based sensors due to their target wave propagation properties. In the literature, several materials are proposed for the substrate, such as lead zirconate titanate (PZT) and LiNbO3. LiNbO3 is capable of generating Rayleigh waves, and it has a high electromechanical factor compared to other piezoelectric materials [16,38].

To determine the right parameters for the SAW sensor, different models have been developed to help during the design phase. In the literature, three main models were used to define the main parameters for SAW devices, which are the impulse response model, coupling-of-modes (COM) theory, the equivalent circuit model, such as Masons model, and finally, the transmission matrix approach. In this work, the impulse response model was applied due to its having the best results in less computational time. Moreover, with piezoelectric materials, the impulse response provides the transmissibility plot. This study analyses the frequency response (with FFT) to optimise the operating frequency because of electromechanical coupling coefficients for a lithium niobate substrate at 872 Mhz. This operating frequency is then validated using finite element analysis for the geometry and material properties defined for the sensor.

### Impulse Response

Impulse response helps to identify the mechanical and electrical behaviour of the SAW device made on the piezoelectric substrate. Many researchers have used this approach for SAW sensor modelling [32,33]. The impulse response model gives the first-order approach to modelling the sensor. The model focuses on determining the IDT parameters using the IDT finger location and the signal generated from them, and this method utilises the fast Fourier transform (FFT) to define the necessary specifications. Further, the model provides good results with less computational time. The model calculates the total energy transfer, radiation conductance *G*(*f*) and susceptance *B*(*f*) of the SAW device. However, the model ignores other effects (such as reflections). It also assumes a constant width for all IDT fingers.

The impulse response *h*(*t*) for a SAW sensor can be defined in the time domain as follows, and the corresponding Fourier transformation of the impulse response is shown as *H*(*f*) [32,34]:

(2) $$\;h\left(t\right)=\{\begin{array}{c} \begin{array}{cc} f_{0}^{3/2}4K^{2}\sqrt{C_{s}}sin\omega_{0}t & \left(0\leq t\leq \frac{N}{f_{0}}\right) \end{array}\\ \begin{array}{cc} 0 & \left(t\langle 0,\;t\rangle \frac{N}{f_{0}}\right) \end{array} \end{array}$$

(3) $$H\left(f\right)=4K^{2}C_{s}Wf_{0}N^{2}\left(\sin X/X\right)e^{-j\left(\frac{N+D}{f_{0}}\right)}$$

(4) $$X=N\pi \left(\frac{f-f_{0}}{f_{0}}\right)$$

where $K^{2}$ is the electromechanical coupling coefficient for surface waves, $C_{s}\;$is the capacitance per finger pair, *W* is the finger aperture, and *D* is the distance between IDTs. The total admittance *Y*(*f*) is defined as the combination of radiation conductance *G*(*f*) and susceptance *B*(*f*), defined as:

(5) $$G\left(f\right)=8K^{2}C_{s}Wf_{0}N^{2}{\left|\frac{\sin X}{X}\right|}^{2}$$

(6) $$B\left(f\right)=G\left(f_{0}\right)\left(\frac{\sin\left(2X\right)-2X}{2X^{2}}\right)$$

(7) $$Y=G\left(f\right)+j\left(2\pi fC_{s}+B\left(f\right)\right)$$

The total admittance for the model is important, as it ensures the maximum current flow and the signal matching with the antenna or the electronic feeding system to avoid losses and reflection. The also model calculates the insertion loss, which determines the losses in the IDT design. Insertion loss is calculated by taking the ratio of the power delivered before and after the SAW device is instated between the source and load. It can be described mathematically, as shown below, where *R* is the load resistance:

(8) $$\;\;\;\;\;\;\;\;\;\;\;\;\;\;\;\;\;\;\;\;\;\;\;\;\;IL\left(f\right)=-10\log\frac{2G\left(f\right)R}{{\left(1+G\left(f\right)R\right)}^{2}+{\left(R\left(2\pi fC_{s}+B\left(f\right)\right)\right)}^{2}}$$

An in-house code implemented on MATLAB has been used to calculate the impulse model for the SAW sensor. The initial design needs to operate at 872 MHz. The reason for selecting this frequency is the ability to use more common RF interrogator units. Further, the sensor needs to be small, because the space is limited inside a hip implant (in the proximal zone, length: 72.05 mm, minimum width: 14.72 mm). Therefore, a small number of IDT finger pairs is required while maintaining good performance. The higher the recentre frequency, the higher the sensitivity the sensor can provide. Aluminium is selected as an IDT material due to its low resistivity and cost, as mentioned earlier. Table 1 shows the initial parameters for the proposed two-port SAW device.

**Figure 3:**
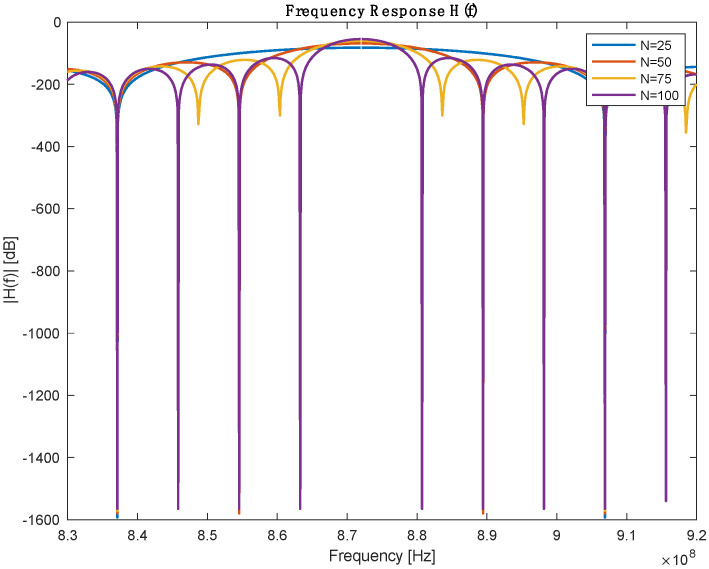
Frequency response with different numbers of IDT pairs.

From the previous table, the number of IDT finger pairs is the only assumed parameter in this table; the rest of the values are defined either by design needs or based on the selected Piezoelectric substance. IDT pairs define the bandwidth of the sensor, but it is worth mentioning that increasing the number of pairs will increase the length of the SAW sensor. A parameter sweeping was performed to select a reasonable IDT finger pair. Figure 3 shows the frequency response for a different number of pairs. Table 2 describes the bandwidth at which 3dB attenuation was observed from the operating frequency for different IDT numbers. 

**Figure 4:**
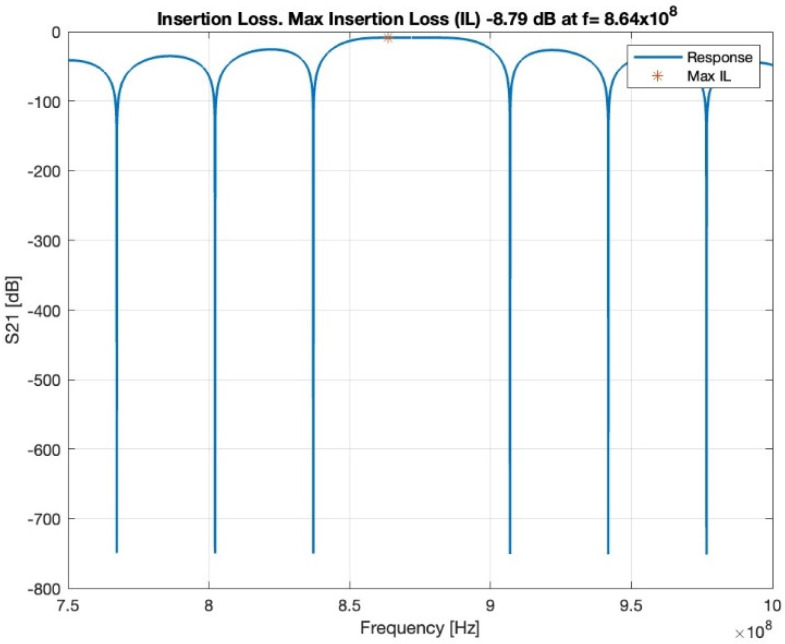
Insertion loss, red star shows the maximum insertion loss.

In the first-order model, the insertion loss achieved a loss of −8.79 dB at 864 MHz, as shown in Figure 4. The loss value is related to the operating frequency.

**Figure 5:**
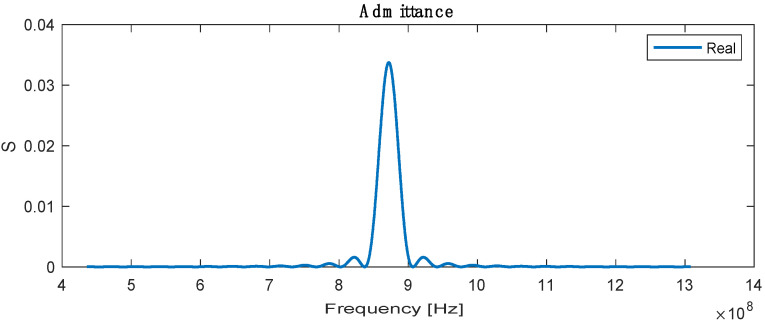
Real admittance for SAW sensor.

The admittance is the sum or superposition of the conductance and susceptance, defined as the ratio of the input current to voltage, and the highest admittance is recorded at a resonance frequency with a value of 0.032 S, as shown in Figure 5.

## 3. Finite Element Analysis (FEA) of SAW Sensor

### 3.1. Electrical Potential of SAW Sensor

**Figure 6:**
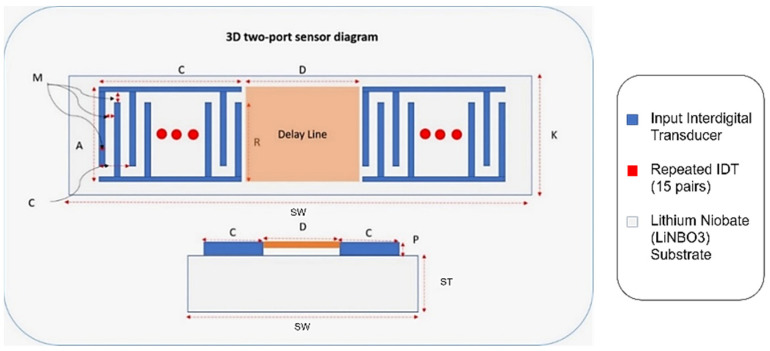
Two-port SAW sensor with design parameters.

A finite element 3D model of a two-port SAW sensor is developed in COMSOL Multiphysics, as it provides a decent working interface for piezoelectric study (Figure 6). COMSOL allows the platform to work in a simulation environment in which multiple physics are used for the desired study for the simulation models. The working principle of COMSOL is partial differential equations, which are the building blocks of scientific phenomena and the coupling of Multiphysics. The operating frequency of the chosen SAW device is 872 MHz with 15 IDT pairs, and the Rayleigh velocity for the LiNbO3 substrate is 3488 m/s. Supplementary Table S1 shows the design parameters of the two-port SAW sensor.

Correspondingly, Figure 6 demonstrates an annotated diagram of the SAW sensor model with the parameters. The SAW device simulation structure is shown in Supplementary Figure S1. The dimensions of the device follow the design parameter, as shown in Table 3. Materials added in the simulation model include aluminium and LiNbO3 for the IDT and substrate, respectively. The material properties of aluminium are taken from the built-in COMSOL library, whereas the material for the substrate is YZ-cut LiNbO3. The critical material properties of the SAW device are shown in Supplementary Table S2.

**Figure 7:**
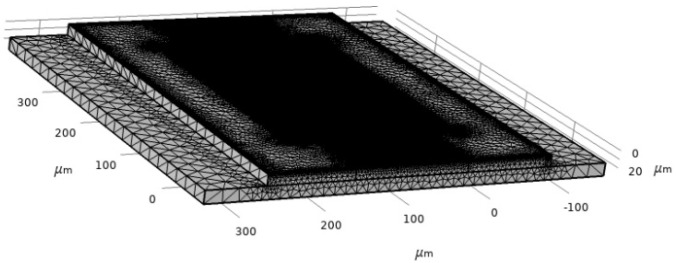
Two-port SAW sensor mesh.

SAW characteristics are determined by using a piezoelectric device interface module, which is the Multiphysics module of the Piezoelectric effect coupled with solid mechanics and electrostatics. The boundary condition is the most critical part of the simulation to define the model. For this model, structural and electrical boundaries are implemented. The bottom of the SAW device is assigned as a fixed constraint since the surface wave is assumed to disappear upon moving two or three wavelengths towards the bottom. The left and right sides of the device are assumed to be periodic boundaries. The periodic boundary signifies the same value of electric potential and displacement across the desired boundary of the model. The rest of the sides of the device are free from constraints. The meshing of the device is performed by physics-controlled mesh with the preassigned setting of the element at extra fine (Figure 7). The mesh consists of 625,937 domain elements, 161,930 boundary elements and 52,214 edge elements.

### 3.2. Modal Analysis of SAW Sensor

Modal analysis of the SAW device is used to analyze the value of the eigenfrequency of the SAW device and further verify whether the given model is operated at the required frequency. The 3D model is used for simulation to find the Eigen frequency of the FE model. A fixed boundary is applied to the base of the device; the substrate will be bonded to the hip implant from the bottom of the substrate. In COMSOL Multiphysics, the simulation showed that the eigenfrequency is equal to 872 MHz. The result of the Eigen frequency comes to be the same as the device design frequency (872 MHz), which verifies the design of the model and the parameters used in the numerical study. This adopted boundary was used in COMSOL Multiphysics software to validate the operating frequency in the numerical study to be effective at 872 MHz with 50 ohms impedance. The stationary analysis provided the additional response of the Multiphysics coupling. In the ANSYS software, the same boundary was adopted when used on the hip implant (fixed base). The strain exerted on the sensor was analysed in terms of voltage output and compared with values in the literature to demonstrate its applicability in cementless applications of hip implants.

### 3.3. Frequency Analysis

**Figure 8:**
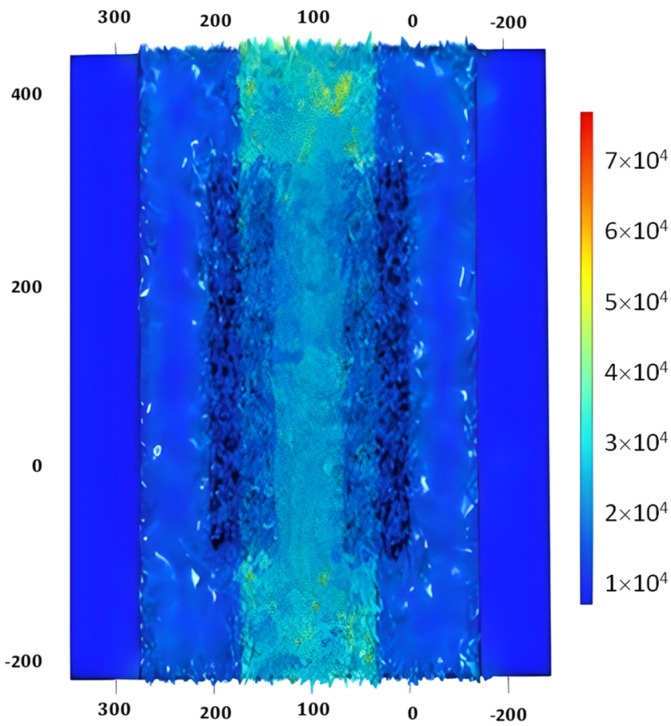
S11 parameter of SAW device.

To find the scattering parameter of the SAW device, the ‘Terminal’ feature is used, which is a predefined feature in COMSOL Multiphysics. By applying the ‘Terminal’ feature, the scattering parameter is calculated automatically by the solver irrespective of the terminal numbers. The impedance is taken to be 50 ohms. Further, the study chosen is ‘frequency’, and the range of frequency for evaluation is 867 MHz to 877 MHz. The S11 parameter is extracted and is shown in Figure 8. The S11 parameter is the rate of reflected and incident power based on the acoustoelectric and wave reflection properties of the SAW material [41].

### 3.4. Stationary Analysis of SAW Sensor

**Figure 9:**
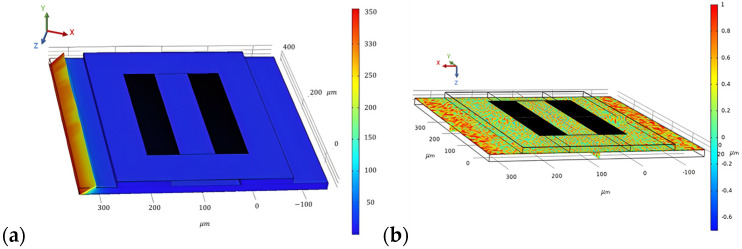
Surface von Mises stress distribution (**a**) and first principal strain on the SAW device for a force of 200 N/m^2^ applied at the steel beam (**b**).

To evaluate the stationary analysis, the Piezoelectric effect is coupled with solid mechanics; electrostatics is removed, and only solid mechanics are considered. A pressure of 200 N/m^2^ is applied at the boundary of the steel beam on which the SAW sensor is placed. The stationary study is computed, and the results are generated for the stress distribution, as shown in Figure 9a. Correspondingly, the result for the first principal strain on the SAW device is generated, which is shown in Figure 9b.

### 3.5. Performance Extension for Cantilever

The beam connected to the sensor utilises a piezoelectric patch that is subjected to loads. The voltage output generated across the piezoelectric patch can be compared using the voltage output *V*(*t*) generated across the patch, such that the equation used can be modelled as the following:

(9) $$\frac{V\left(t\right)}{R_{l}}+C^{s}\dot{V}\left(t\right)-\Theta \dot{x}\left(t\right)=0$$

Here, $\dot{x}\left(t\right)$ is the velocity of the deformation from the load, and $R_{l}\;$is the load resistance applied across the sensor. Further, Θ and $C^{s}$ are the electromechanical coupling coefficient and the clamped capacitance, both of which are properties of the piezoelectric element modelled, respectively.

The structural response of the static and dynamic model was performed on ANSYS Academic 2022R2. The piezoelectric modelling of the SAW sensor was performed using the piezoelectric solver extension. The electromechanical coupling between strain and voltage output is shown in the Supplementary Table S2. The results of the simulation were compared to previous work with modelling ANSYS with piezoelectric elements and the author’s experimental data with good initial convergence [42,43,44]. The constitutive equation of piezoelectric materials using a stress-charge form is expressed as:

(10) $$\left\{\begin{array}{c} \left\{T\right\}\\ \left\{D\right\} \end{array}\right\}=\left[\begin{array}{cc} \left[c^{E}\right] & \left[e\right]\\ {\left[e\right]}^{T} & -\left[\varepsilon^{S}\right] \end{array}\right]\left\{\begin{array}{c} \left\{S\right\}\\ \left\{E\right\} \end{array}\right\}$$

where {*T*} represents the stress, I is the electric flux density, $\left[c^{E}\right]$ is the elasticity at a constant electric field, [e] is the piezoelectric stress, $\left[\varepsilon^{S}\right]$ is the dielectric matrix at constant mechanical strain, {*S*} is the elastic strain vector, and {*E*} is the electric field intensity vector. The material properties of the lithium niobate material are shown in the Supplementary Materials (Table S2).

## 4. Finite Element Analysis (FEA) of Hip Implant

### 4.1. Simulation Setup

The proposed hip implant was modelled based on a Summit Hip implant model that is readily available in the market and utilised in prior literature. An additional stage of the hip bone is used, and an ACME screw thread is on the ball joint. The hip implant uses a titanium (Ti-6Al-4V) alloy, which has been demonstrated in the literature as providing excellent strength to dynamic loading and resistance to the antibodies and is therefore among the most frequently used materials in joint prostheses according to the standard of the American Society for Testing and Materials (ASTM). The ball joint casing for hip prosthesis uses polyethene material settings. Table 3 shows the material properties of the bone and implant used.

**Figure 10:**
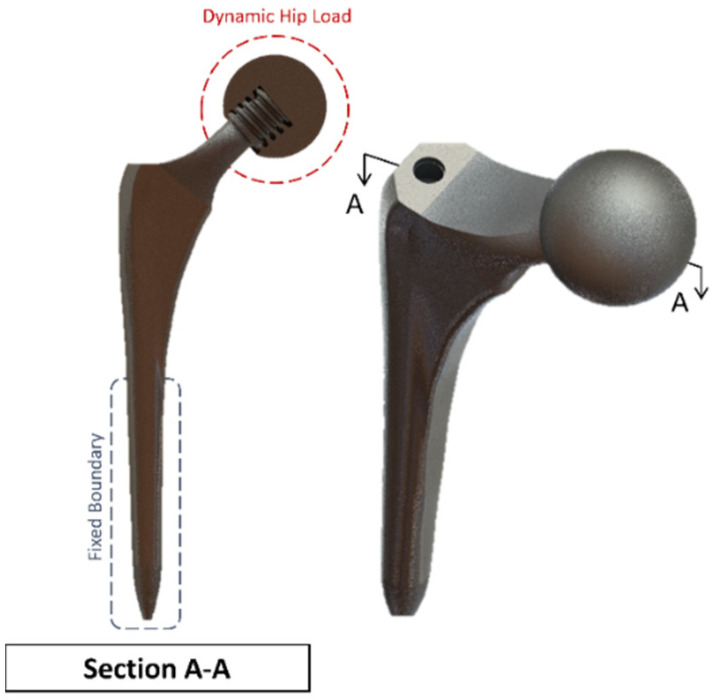
Summit Hip implant model.

The femur bone is assumed to have an isotropic linear behaviour with bone properties adopted from the literature [29,30]. Ideally, a combination of elastic, hyper-elastic, elastic-plastic and anisotropic material properties would provide a more realistic representation but would be computationally prohibitive for the current Multiphysics investigation [25]. The hip implant that is used (as shown in Figure 10) with the cross-section makes the screw thread visible. It is modelled with a ball and socket joint to biomechanically replicate hip stability in a total hip replacement surgery due to damage in the cartilage and bone. The cup is tailored for each patient individually and is custom-made for each patient. In this model, a sample dimension was used based on the values obtained from the literature and in vivo studies [45]. Different stem design shapes have been investigated in the literature, with circular and trapezoidal-shaped stems being shown to have lower von Mises stress concentration compared to elliptical and oval shapes [46].

A clamped boundary condition is attached towards the bottom of the implant that sits within the femur bone. Meanwhile, axial and moment forces were applied at the ball joint of the hip implant to simulate the static loading conditions when attached to the body. Two positions were outlined for potential SAW sensor positioning, with the positions labelled as A and B, respectively. These are placed further away from the ball joint on a flatter surface of the implant to ensure that the strain from the loading does not induce any piezoelectric effects that can generate a significant signal from the electromechanical coupling. In femur simulations, a comprehensive simulation utilizing different loading conditions and complete boundaries is difficult because of the non-uniform shape of the bone concerning the implant. As a result, various boundary conditions and simulated loading conditions have been adopted with their approximation and results. A stringent mesh sensitivity analysis was performed for deformation and stress convergence in preliminary studies, as shown in Table 4. In this study, the femur is simulated using three different boundary and loading conditions, as shown in Table 5 for Femur A, B and C for the different conditions, respectively.

### 4.2. Sensitivity Analysis

To minimise the effect of mesh influence on the finite element analysis, a mesh sensitivity study was first performed on the Summit hip implant model in Figure 10 using the first loading and boundary conditions highlighted in Table 5 for the simulated scenario of upper-body loading. The results of the sensitivity study are shown in Table 4. For the first two meshes, the element sizing was set to default, and the resolution of the mesh was increased. For Mesh 3 to Mesh 7, different sizings were applied throughout the body. The results of average stress in Table 4 clearly indicate that mesh convergence starts for element sizing less than 2.80 mm, and therefore Mesh 3 onwards can be used. 

### 4.3. Results of FEA of Hip Implant

**Figure 11:**
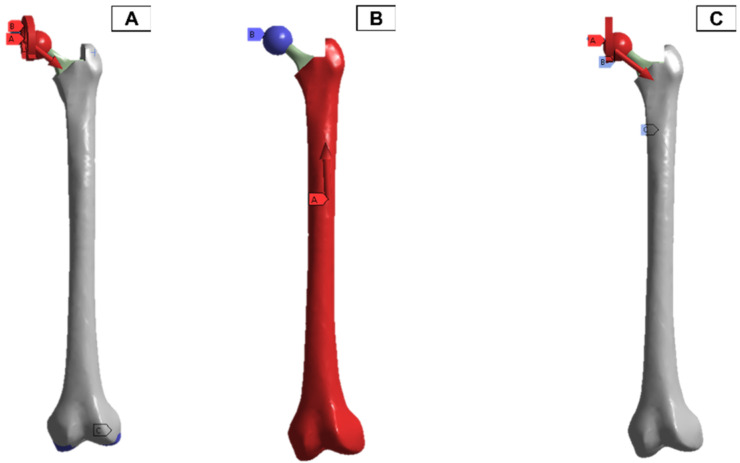
Femur simulation boundary condition setup. Red: applied surface load, blue: fixed boundary.

In the finite element model setup, a mesh sizing of 2.7 mm was adopted, and the output was realised for a convergence in the stress variables for the static loading conditions. The loading and boundary conditions adopted were based on in-vitro studies in the literature. As a result, there are different force directions and magnitudes due to the upper- and lower-body loading. These are shown in Figure 11, where A and C have the force and moment applied from the hip joint, and B has the force applied from the lower body.

**Figure 12:**
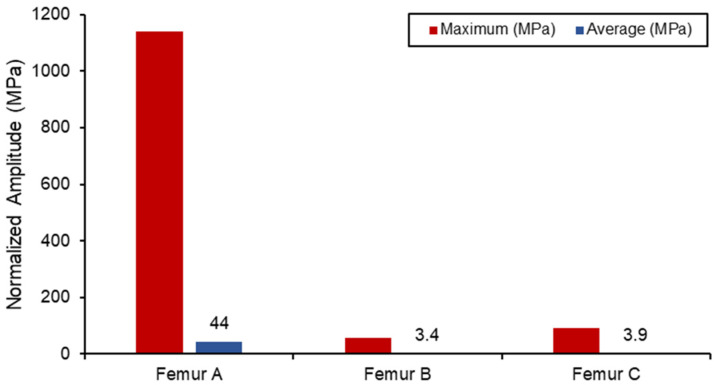
Von Mises stress loading in femur simulation.

The static simulation of the different loadings was run, with the average and maximum stress values shown in Figure 12. All the results with the femur were within proximity to values found in the literature for similar loading conditions, with differences observed only due to different geometries. Due to the relatively small boundary fixture, femur (A) experienced the largest stress concentration as the bone acted like a cantilever setup. As a result, SAW sensor simulation readings would be skewed due to the larger bending deformation. The femur (B) has the lowest stress concentration in comparison also due to the lower loading conditions from the lower body. Both femur (B) and femur (C) loading do not exceed the yield tensile or compression strengths of the bone material used [47,48].

**Figure 13:**
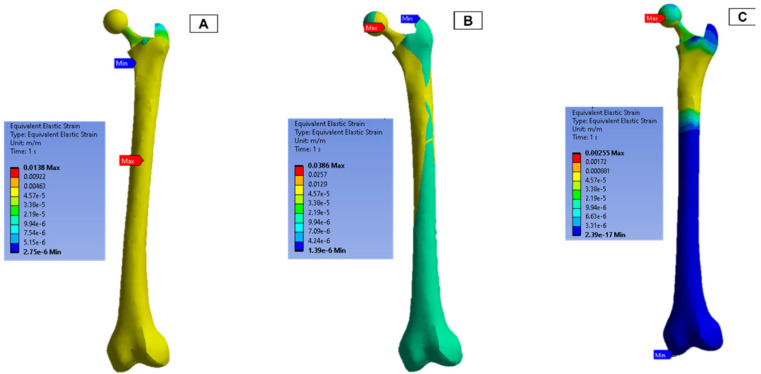
Femur simulation strain concentration results.

The strain measurements of the femur are shown in Figure 13. In both femur (B) and (C), the upper half of the femur experienced a higher strain concentration, and the maximum concentration was around the ball joint.

### 4.4. Integration of Hip Implant with SAW Sensor

**Figure 14:**
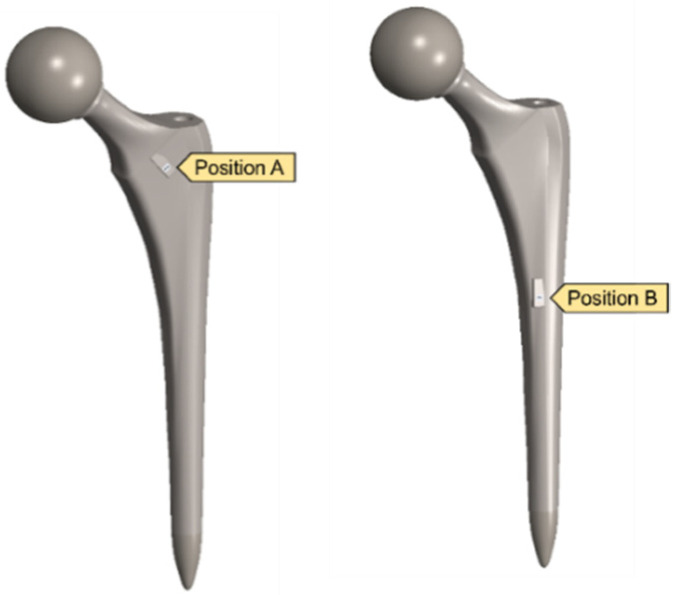
Implanted SAW sensor on hip implant.

After validating both the sensor and hip implant individually, the work was extended to combine both systems to test the feasibility of THR applications. Figure 14 highlights the SAW sensor used in the hip implant at two different locations.

Traditionally, the application of the SAW sensor is to relay the information and status of the implant, but in vivo studies with piezoelectric materials have shown that the sensor can be utilised as the energy and detection unit when attached to an implant [49]. Because the hip implant model was significantly larger than that of the SAW sensor, a mesh refinement sizing was added for the SAW model and in the surrounding region.

**Figure 15:**
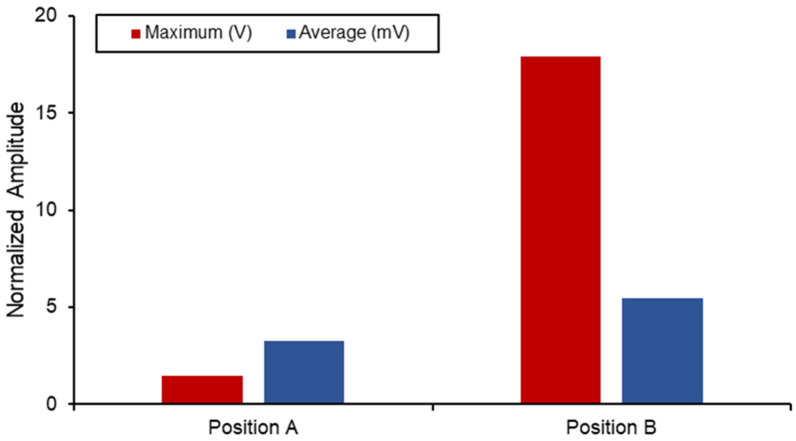
SAW sensor voltage output in hip implant.

Figure 15 displays a comparison between the different voltage output out of the SAW sensors when subjected to the combined static load at the ball joint. Position B naturally has a higher output and experienced strain due to its proximity to the fixed boundary. However, when comparing the differences in the average measurements between the two, a 40% increase was seen in position B. The voltage output produced by the implants in this position is higher than the values reported in the literature, and the results are shown in Table 6. However, this degree of sensitivity and proximity to the fixed joint can introduce noise and interference with the electromechanical properties of the sensor as well as the acoustics. Therefore, additional work on the vibro-acoustics of wave propagation with the involvement of dynamic loading would address the applications of long-term monitoring implant systems.

In the literature, most hip implant-loosening detection systems utilise temperature, force, acoustic emission, vibration, and strain gauge sensors. Table 7 compares the existing hip implant-loosening detection system to the proposed SAW sensor-based system. The temperature and strain-based implant-loosening detection device use inductive power. Due to telemetry circuit heat and eddy currents, high power consumption might affect temperature measurements. In a vibration and acoustic emission-based system, loosening was identified by measuring the output signal’s amplitude using a fast Fourier transform (FFT) analysis, where inefficient signal transmission and signal dampening lead to poorer system efficiency. The proposed SAW sensor system is powered by itself, and the induced voltage is utilised as the indicator to detect implant loosening. In conclusion, it can be said that the proposed system can be a possible solution to avoid the limitations of existing systems. 

This article enriches the available literature on SAW sensors and hip implants by providing the results of analytical, numerical and computational work, with validation from each step. Additionally, there has been no universally adopted framework for FEA, as the methods in the literature vary with respect to the loading and boundary conditions. In this article, we combined the three most common configurations for loading and boundary conditions and compared the results of all three with those found in literature. In addition, this study helps in improving the robust results associated with SAW sensors in total hip replacements. The results presented in Table 6 highlight the improved selection of LiNbO_3_ substrates for sensitive and self-sustaining SAW sensors in future works. Moreover, the comparison highlighted in Table 7 suggests that the lower power requirements and higher operating frequency mean that the sensor is more resistant to shock at lower frequencies associated with joint movements.

## 5. Conclusions

In the current study, the development of a surface acoustic wave sensor for hip implants was studied using numerical and finite element simulations. The results highlighted the characteristics of the SAW sensor in electrical admittance and functionality in the MHz operating frequency. Furthermore, the acoustic emission behaviour was investigated for a lithium niobate material and a two-port sensor. The sensor was then studied using a finite element approach starting from its electromechanical properties at 872 MHz. Afterwards, a femur hip bone was modelled with a hip implant for a total hip replacement study. The results were validated against literature values for different loading conditions. The SAW sensor was then mounted on two different positions on the hip implant and statically simulated to measure the response. The voltage output results showed that the SAW sensor could be mounted in the total hip implant for implant-loosening sensing applications. A deeper investigation into the effect of dynamic loading with joint reaction forces and moments can provide a deeper understanding of the feasibility of its use and its effect on acoustic properties and predict the remaining life of the implant for elderly patients.

## Figures and Tables

**Table 1:** SAW device specification.

Parameter	Value
Operating frequency $f_{0}$	872 MHz
IDT finger pairs N	25
Piezoelectric substance	Lithium niobate (LiNbO_3_)
Acoustic velocity in the medium v	3800 m/s
$\mathrm{Coupling}\;\mathrm{coefficient}\;K^{2}$	0.053
Load resistance R	50 Ω
Electrode pair capacitance per unit length	0.55 pf/cm

**Table 2:** Performance comparison between different IDR pairs.

N	Bandwidth (MHz)	IDT Length (μm)
25	69	54.4
50	35	108.9
75	23	163.4
100	17	217.8

**Table 3:** Material properties of the bone and implant in THR [38,39,40].

Material Properties	Cortical Bone	Titanium Alloy	Polyethene
**Density (kg/m^3^)**	1700	4429	950
**Young’s Modulus (GPa)**	11	111	1.1
**Poisson’s Ratio**	0.25	0.339	0.42
**Ultimate Tensile Strength (MPa)**	100	-	33
**Ultimate Compressive Strength (MPa)**	100	-	-

**Table 4:** Mesh sensitivity analysis study on Summit Hip implant.

Mesh Resolution	Element Sizing (mm)	Element Count	Average Mesh Quality	Max. Stress (MPa)	Avg. Stress (MPa)
Mesh 1	-	3129	0.51	163.2	4.34
Mesh 2	-	5133	0.64	283.5	6.42
Mesh 3	2.80	16,947	0.76	304.3	8.76
Mesh 4	2.45	21,099	0.76	308.7	9.02
Mesh 5	2.00	31,728	0.76	298.1	8.96
Mesh 6	1.70	43,188	0.77	328.1	9.14
Mesh 7	1.40	67,677	0.77	358.9	9.22

**Table 5:** Femur Simulation Setup.

Loading Forces	Boundary Type	Simulated Scenario	Setup
1270N Axial Load (Ball Head) 1.13N mm Torsional Load (Ball Head)	Hip implant fix bounded	Upper-body loading and leg extension	Figure 11a
600N Axial Load (Body Shaft)	Ball head fix bounded	Lower-body loading	Figure 11b
1270N Axial Load (Ball Head) 1.13N mm Torsional Load (Ball Head)	Femur tip fix bounded along medial condyle and lateral Condyle	Upper-body loading and leg extension	Figure 11c

**Table 6:** Energy-harvesting performance of hip implant.

	Current Work (2022)	Lange & Kluess (2021) [50]	Lange et al. (2020) [51]
**Substrate Material**	LiNbO_3_	PZT Ceramic	
**Piezoelectric Young’s Modulus (GPa)**	70	52.4	
**Maximum Voltage Output (V)**	17.88	7.64 *	2.88 *

* Reported values estimated through impedance matching the resistance value.

**Table 7:** Comparison of existing hip implant-loosening detection system to the proposed SAW sensor-based system.

Reference	Monitoring Method	Implant Type	Operating Frequency
Bergmann et al. [25]	Temperature	Cementless	N/A
Graichen et al. [52]	Temperature and force	Cementless	47 to 220 MHz
Marschner et al. [28]	Vibration	N/A	125 KHz
Ruther et al. [53]	Vibration	N/A	N/A
Burton et al. [30]	Strain	N/A	10 to 14 MHz
Rodgers et al. [26]	Acoustic Emission	Cement/Cementless	N/A
Current Work	SAW Sensor	Cementless	872 MHz

## Data Availability

Not applicable.

## Supplementary Materials

The following supporting information can be downloaded at: https://www.mdpi.com/article/10.3390/bios13010079/s1, Table S1: SAW sensor design parameters; Figure S1: COMSOL model of a two-port SAW Sensor; Table S2: Material Properties of SAW Sensor.
