# Supplementary material for: Surface Acoustic Wave (SAW) Sensors for Hip Implant: A Numerical and Computational Feasibility Investigation Using Finite Element Methods
Source: Biosensors (Basel). 2023 Jan 2;13(1):79. doi: 10.3390/bios13010079 (PMC9855817; doi:10.3390/bios13010079)
Supplement: Supplementary file 1 [file biosensors-13-00079-s001.zip › biosensors-2010927-supplementary.pdf]

Supplementary Materials

Supplementary Table S1. SAW sensor design parameters

Design Parameters	Value
**Frequency (f)**	872 [MHz]
**Velocity (v)**	3488 [m/s]
**IDT Numbers (IDT)**	15
**Single Finger Width/Distance between fingers (M)**	1.08945E-6 [m]
**Single Finger Thickness (P)**	2.1789E-07 [m]
**Substrate Thickness (ST)**	20E-06 [m]
**Substrate Width (SW)**	435.78E-06 [m]
**Aperture (R)**	0.000305046 [m]
**IDT Length (C)**	6.5367E-05 [m]
**IDT Width (A)**	0.000309E-04 [m]
**Outer Length (OL)**	7.321100917E-04 [m]
**Finger Length (L)**	R-M
**Width of IDT Pair (WIP)**	M×4
**Delay Line Length**	8.715596E-4 [m]

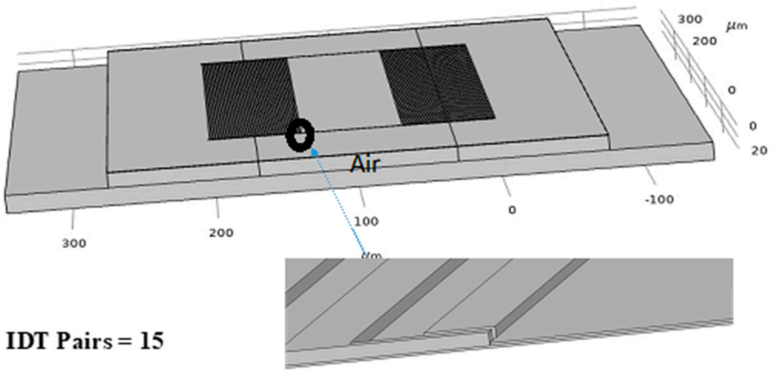

Supplementary Figure S1. COMSOL model of a two-port SAW Sensor.

Supplementary Table S2. Material Properties of SAW Sensor

Materials	Description	Value
**Aluminium**	Relative permittivity	1
	Density (kg/m^3)	2700
	Young's modulus [Pa]	7.00E+10
	Poisson's ratio	0.33

	Density (kg/m^3)	4647
**Lithium Niobate**	Elastic constants (GPa)	$\begin{bmatrix} 242.4 & 75.2 & 75.2 & 0 & 0 & 0 \\ 75.2 & 203 & 57.3 & 0 & 8.5 & 0 \\ 75.2 & 57.3 & 203 & 0 & -8.5 & 0 \\ 0 & 0 & 0 & 75.2 & 0 & 8.5 \\ 0 & 8.5 & -8.5 & 0 & 59.5 & 0 \\ 0 & 0 & 0 & 8.5 & 0 & 59.5 \end{bmatrix}$
	Piezoelectric constants (C/m^2)	$\begin{bmatrix} 1.33 & 0.23 & 0.23 & 0 & 0 & 0 \\ 0 & 0 & 0 & -2.5 & 0 & 3.7 \\ 0 & -2.5 & 2.5 & 0 & 3.7 & 0 \end{bmatrix}$
	Dielectric constants (ε_0)	$\begin{bmatrix} 28.7 & 0 & 0 \\ 0 & 85.2 & 0 \\ 0 & 0 & 85.2 \end{bmatrix}$
